# Gambling problems among patients in primary care: a cross-sectional study of general practices

**DOI:** 10.3399/bjgp17X689905

**Published:** 2017-03-14

**Authors:** Sean Cowlishaw, Lone Gale, Alison Gregory, Jim McCambridge, David Kessler

**Affiliations:** Centre for Academic Primary Care, School of Social and Community Medicine, University of Bristol, Bristol, UK; visiting fellow, Centre for Gambling Research, ANU Centre for Social Research and Methods, Research School of Social Sciences, Australian National University (ANU), Canberra, Australia.; Centre for Academic Primary Care, School of Social and Community Medicine, University of Bristol, Bristol, UK.; Centre for Academic Primary Care, School of Social and Community Medicine, University of Bristol, Bristol, UK.; Professor of Addictive Behaviours and Public Health, Department of Health Sciences, University of York, York0, UK.; Centre for Academic Primary Care, School of Social and Community Medicine, University of Bristol, Bristol, UK.

**Keywords:** cross-sectional study, England, gambling, general practice

## Abstract

**Background:**

Primary care is an important context for addressing health-related behaviours, and may provide a setting for identification of gambling problems.

**Aim:**

To indicate the extent of gambling problems among patients attending general practices, and explore settings or patient groups that experience heightened vulnerability.

**Design and setting:**

Cross-sectional study of patients attending 11 general practices in Bristol, South West England.

**Method:**

Adult patients (*n* = 1058) were recruited from waiting rooms of practices that were sampled on the basis of population characteristics. Patients completed anonymous questionnaires comprising measures of mental health problems (for example, depression) and addictive behaviours (for example, risky alcohol use). The Problem Gambling Severity Index (PGSI) measured gambling problems, along with a single-item measure of gambling problems among family members. Estimates of extent and variability according to practice and patient characteristics were produced.

**Results:**

There were 0.9% of all patients exhibiting problem gambling (PGSI ≥5), and 4.3% reporting problems that were low to moderate in severity (PGSI 1–4). Around 7% of patients reported gambling problems among family members. Further analyses indicated that rates of any gambling problems (PGSI ≥1) were higher among males and young adults, and more tentatively, within a student healthcare setting. They were also elevated among patients exhibiting drug use, risky alcohol use, and depression.

**Conclusion:**

There is need for improved understanding of the burden of, and responses to, patients with gambling problems in general practices, and new strategies to increase identification to facilitate improved care and early intervention.

## INTRODUCTION

Participation in gambling is increasing in the UK, with surveys indicating that around 59% of British adults reported gambling activities (excluding National Lottery) in 2010, which was an increase of 7% from 2007.^1^ These trends have occurred in the context of developments in gambling technologies (such as electronic gambling machines and online gambling) and increased exposure (for example, gambling-related advertisements grew by almost 500% between 2007 and 2012),^2^ and larger numbers of people experiencing problems with gambling.^1^ These problems encompass a spectrum of difficulties that are defined mainly by gambling-related harms (for example financial crises or relationship breakdown),^3^ and can sometimes reach levels of severity that warrant diagnoses of pathological gambling or gambling disorder (in the ICD-10 4 and DSM-5,^5^ respectively). Prevalence studies indicate that around 7% of males (2% of females) experience at least some problems with gambling annually in the UK, with higher levels among young adults (for example, 17% of males aged 16–24 years reported at least some problems in 2012).^6^ There is also a socioeconomic gradient of risk, whereby elevated risk of gambling problems is associated with low income and high deprivation.^7^

Gambling problems cluster with other health-related behaviours,^8^ and are associated with anxiety disorders and psychosomatic complaints, and high rates of suicidal ideation and attempts.^9^ These problems are also associated with overuse of healthcare services, with problem gamblers being twice as likely to consult their GP for mental health concerns, five times as likely to be hospital inpatients, and eight times as likely to access psychological counselling, when compared with people with no such problems.^9^ Help seeking for gambling is uncommon, however, and usually crisis-driven,^10^ and thus occurs only after experiencing severe gambling-related harms. Accordingly, there is a strong need for initiatives to increase help seeking and early intervention, and these include new means of identification and response within generalist healthcare settings.

Primary care is an established context for addressing health-related behaviours (for example, alcohol misuse),^11^ and may be an important setting for identification of problematic gambling.^12^ High use of services^9^ suggests overrepresentation of gambling problems in primary care, and particularly within practices that serve vulnerable populations. This is supported by US data suggesting rates of gambling disorders ranging from 6%^13^ to 15%^14^ among primary care attenders (relative to estimates from population-based studies that range from 0.2% to 1.0%),^15^ and higher levels within low income populations.^14^ It is already recommended that UK GPs screen high-risk groups (for example, those reporting financial problems), and refer cases for specialist treatment.^12^ This is notwithstanding the lack of any evaluation of gambling problems in UK general practices, whereby the prevalence of conditions remains unknown. In this context, the current study aimed to:

- provide data on the extent of gambling problems among patients attending general practices in England; and
- explore variability according to practice and patient characteristics, and thus indicate clinical settings or patient groups experiencing heightened vulnerability.

**How this fits in**

Gambling problems are emerging concerns for public health in the UK, and primary care may be a context for identifying patients who would benefit from intervention or specialist services. However, there are no relevant data on UK general practices, and this study assessed the extent of gambling problems, and patient groups that may be vulnerable. The findings suggest around 1 in 20 patients report gambling problems, which were mostly of low to moderate severity, in routine general practice. They highlight the need for increased acknowledgement and capacities to respond to patients with gambling problems in general practices.

## METHOD

### Participants and procedure

The target population comprised patients attending general practices in the Bristol region of south west England. Eleven practices were purposively sampled according to population deprivation and patient characteristics, as follows:

- deprivation levels were quantified using data from the Office for National Statistics, which indicated four practices from deprived areas (top 30% for deprivation in England), two practices in areas of low deprivation (bottom 30%), and three practices in a moderate band (middle 40% for deprivation); and
- two practices were not sampled on the basis of population deprivation as measured by ONS data. One was a student health service and one a practice for the homeless. The latter were targeted to assess risk according to key population subgroups.

Patients aged ≥18 years and attending practices for any reason were eligible to take part in the study, but were excluded if they were unable to understand English sufficiently, required immediate medical attention, or were unable to give consent. Patients were approached by a researcher in waiting rooms before appointments, and were provided with information about the study. Those who provided consent were given anonymous questionnaires. These were self-completed and returned in the waiting room or using pre-paid envelopes, and yielded *n* = 1058 questionnaires. Across practices sampled according to deprivation, the patient numbers ranged from *n* = 58 to *n* = 122. There were *n* = 17 and *n =* 163 participants recruited from the practice for patients who are homeless and the student health service, respectively. Sociodemographic characteristics are shown in Table 1.

**Table 1.** Sample sociodemographic characteristics of patients (*n* = 1058)

	***n***	**%**
Sex (female)	636	64.7

Age, years		
18–24	211	20.7
25–34	154	15.1
35–44	137	13.4
45–64	284	27.8
≥65	235	23.0

Relationship status		
Single (never married)	341	33.3
Married/living with partner	526	51.4
DSW/other	156	15.2

Education		
Secondary school or less	270	27.0
Post-secondary school education	627	62.6
Postgraduate education	60	6.0
Other	44	4.4

Employment		
Employed	398	39.33
Unemployed	126	12.45
Retired	226	22.33
Student	166	16.40
Other	96	9.49

Ethnic group (white)	889	87.67

Small amounts of missing data (< 10% per variable) mean that patient numbers across categories may not aggregate to 100%. There were n = 75 missing values on sex; n = 37 missing values on age; n = 35 missing values on relationship status; n = 57 missing values on education; n = 46 missing values on employment; and n = 44 missing values on ethnicity. DSW = Divorced/separated/widowed.

### Measures

Brief measures identified mental health concerns and addictive behaviours. These included the two-item Whooley scale for depression,^16^ and the GAD-2^17^ scale for anxiety, which are recommended in primary care.^18^ Risky alcohol use was measured using the three consumption items from the Alcohol Use Disorders Identification Test (AUDIT-C).^19^^,^^20^ Non-prescription and recreational drug use was assessed using a Single-Item Screening Question (SISQ) for unhealthy drug use.^21^ The format of this item, which required numeric indications of number of times (in the past year) using an illegal drug or prescription medication for non-medical reasons, was modified and comprised a binary response (yes or no) indicating any past year usage.

Gambling frequency was assessed using items derived from the British Gambling Prevalence Surveys,^1^ asking about purchases of lottery or instant win/scratch tickets, play on bingo, casino table games, slot machines and other electronic gambling machines, games of skill against other individuals, or betting money on sporting events. These items used past year time frames (0 = Never, 6 = ≥4 times a week), along with an item about any other gambling. Patients reporting gambling were then asked to complete the Problem Gambling Severity Index (PGSI),^22^ which consists of nine items scored on 4-point response scales (0 = Never, 3 = Almost always) that relate to past year experiences. The study used a criterion of PGSI ≥5 for problem gambling (which has been shown to yield greatest classification accuracy relative to clinician ratings involving detailed case conceptualisations),^23^ with scores of PGSI 1–4 indicating low to moderate severity problems (given that all such responders were demonstrating at least some signs of problematic gambling). There was a single item about whether family members or close relatives had ever had problems with gambling, which was adapted from epidemiological surveys^24^ and had a binary response format.

### Data analyses

Data-file preparation was conducted using SPSS (version 21), while analyses were conducted using Program R. These comprised descriptive analyses of rates of gambling problems and other mental health concerns and addictive behaviours. Exploratory analyses of variability according to practice characteristics were conducted, followed by evaluations of associations with patient-level characteristics. These comprised Pearson χ^2^ tests and logistic regression models that explored significant effects. The latter specified gambling problems as endogenous variables, and with patient characteristics treated as exogenous. These were evaluated in separate models, which thus estimated bivariate associations through odds ratios (ORs) and 95% confidence intervals (CIs).

## RESULTS

Preliminary analyses indicated modest levels of missing data ranging from around 5% (depression) to 13% (alcohol) across most measures, and were managed through pairwise deletion. There were higher levels for the PGSI, however, with around 45% of eligible participants (that is, reporting gambling in the past year) having missing data across items. Exploratory analyses indicated that around 90% of these patients reported gambling on lottery or with instant win tickets only, and suggested that missing data were attributable mainly to such patients failing to define these activities as gambling. Missing data were addressed using zero-fill techniques, and thus assumed no gambling problems.

Table 2 indicates frequencies of gambling problems and mental health problems or addictive behaviours. There were 0.9% of patients demonstrating problem gambling (PGSI ≥5), and 4.3% exhibiting problems that were low to moderate in severity (PGSI 1–4). Thus, a total of 5.2% of patients (95% CI = 4.0% to 6.8%) exhibited at least some gambling problems across a spectrum of severity. There were 7.2% of patients reporting gambling problems among family members, and this included eight patients reporting problems with their own gambling (PGSI ≥1). Levels were lower than rates of other mental health problems and addictive behaviours.

**Table 2.** Estimates of the extent of mental health problems and addictive behaviours, including gambling problems (*n* = 1058)

	***n***	**%**	**95% CI**
Gambling			
PGSI ≥5	10	0.9	0.5 to 1.8
PGSI 1–4	45	4.3	3.2 to 5.7
Problems in the family with gambling	73	7.2	5.7 to 9.0

Mental health/addictive behaviours			
Depression (Whooley ≥1)	561	55.8	52.7 to 58.9
Anxiety (GAD-2 ≥2)	262	27.0	24.3 to 30.0
Alcohol (AUDIT-C ≥5)	307	32.4	29.4 to 35.5
Drug use (SISQ)	140	14.3	12.2 to 16.7
PGSI ≥1 across practice characteristics			
High deprivation (*k* = 4, *n* = 380)	23	6.1	4.0 to 9.1
Moderate deprivation (*k* = 3, *n* = 331)	13	3.9	2.2 to 6.8
Low deprivation (*k* = 2, *n* = 184)	6	3.3	1.3 to 7.3
Student health service (*k* = 1, *n* = 163)	13	8.0	4.5 to 13.5

*PGSI = Problem Gambling Severity Index. Whooley = Whooley depression scale. GAD-2 = 2-item GAD scale for anxiety. AUDIT-C = 3-item consumption scale from the AUDIT. SISQ = Single-Item Screening Question for Unhealthy Drug Use.* k* = number of practices. For the purposes of these analyses the clinic was combined for patients who are homeless with the highly-deprived practices (thus k = 5).*

Subsequent analyses explored variability in gambling problems (PGSI ≥1) according to practice characteristics. Given small numbers of practices in this study, the results (Table 2) are highly exploratory. They yielded trends (*P* <0.10) suggesting elevated rates in the student health service, however, when compared with practices characterised by low (OR 2.57, 95% CI = 0.99 to 7.47) and moderate deprivation (OR 2.12, 95% CI = 0.95 to 4.73). Modest elevations were observed for highly-deprived practices but were not significantly different when compared with practices characterised by low (OR 1.91, 95% CI = 0.81 to 5.25) or moderate deprivation (OR 1.58, 95% CI = 0.95 to 4.73). These deprived practices included the clinic for patients who are homeless, who were too few for statistical comparison (*n* = 17), but exhibited extremely high rates of gambling problems (29.4%).

Bivariate associations involving any gambling problems (PGSI ≥1) and patient characteristics are shown in Table 3. These indicated significant associations with sex, age, and relationship status. Logistic regression illustrated higher rates in males (compared with females: OR 2.55, 95% CI = 1.44 to 4.55), patients aged 18–24 years (compared with 35–44-year-olds: OR 2.43, 95% CI = 1.21 to 5.06), and patients who were single/never married (compared with married or cohabitating: OR 2.35, 95% CI = 1.32 to 4.29). Exploratory analyses indicated that males aged 18–24 years were a particularly vulnerable group, with 25.4% (95% CI = 15.6% to 38.2%) reporting any gambling problems. Patients screening positive for depression demonstrated a twofold increase in rates of gambling problems (OR 2.08, 95% CI = 1.15 to 3.94), while risky alcohol use was associated with a near threefold increase (OR 2.78, 95% CI = 1.60 to 4.89). Drug use was associated with a fivefold increase in gambling problems (OR 5.03, 95% CI = 2.78 to 8.99).

**Table 3.** Analyses of associations with any gambling problems (PGSI ≥1) and patient-level sociodemographic and clinical characteristics (n = 1058)

		***n***	**%**	**χ^2^**	***P*-value**
**Sociodemographic characteristics**					

Sex	Male	29	8.4	10.0	0.002
	Female	22	3.5		
Age, years	18–24	22	10.4	18.2	0.001
	23–44	8	5.2		
	35–44	4	2.9		
	45–64	13	4.6		
	≥65	5	2.1		
Relationship status	Single, never married	29	8.5	11.9	0.003
	Married/cohabitating	20	3.8		
	DSW/other	4	2.6		
Education	Secondary school or less	9	3.3	2.6	0.272
	Post-secondary school	37	5.9		
	Postgraduate/other	6	5.8		
Employment	Employed	21	5.3	7.7	0.052
	Unemployed	11	8.7		
	Student	11	6.6		
	Retired/other	9	2.8		
Ethnic group	White	45	5.1	0.0	0.969
	Non-white	7	5.6		

**Clinical characteristics**					

Depression	Whooley (≥1)	38	6.8	5.1	0.024
	Whooley (0)	15	3.4		
Anxiety	GAD-2 (≥3)	19	7.3	2.3	0.127
	GAD-2 (<3)	32	4.5		
Alcohol	AUDIT-C (≥5)	30	9.8	12.9	0.000
	AUDIT-C (<5)	24	3.7		
Drug use	SISQ Yes	22	15.7	32.8	0.000
	SISQ No	30	3.6		

*Positive endorsement of either item from the Whooley depression scale was used to indicate possible depression. Scores of* ≥*3 on the GAD-2 were used to indicate potential anxiety. Scores of 5esp on the AUDIT-C were used to indicate high risk (including hazardous and harmful) drinking. DSW = Divorced/separated/widowed. PGSI = Problem Gambling Severity Index. AUDIT = Alcohol Use Disorders Identification Test. SISQ = Single-Item Screening Question for Unhealthy Drug Use.*

## DISCUSSION

### Summary

The results indicated that around 5% of patients report problems with gambling across a spectrum of severity, including approximately 1% who were problem gamblers (PGSI ≥5) and 4% reporting problems that were low to moderate in severity (PGSI 1–4). There were around 7% of patients reporting gambling problems among family members or close relatives, and they were also likely to encounter gambling-related harms.^25^ These rates were lower than other mental health concerns (for example, depression: 56%) and addictive behaviours (for example, risky alcohol use: 32%) that have stronger traditions of recognition in primary care.

Notwithstanding, the study indicated groups and perhaps clinical contexts that were characterised by heightened vulnerability. There were high rates among males and young adults (the extent of any gambling problems among males aged 18–24 years was 25.4%, 95% CI = 15.6% to 38.2%), and more tentatively, within the student healthcare setting. Gambling problems were elevated among patients demonstrating drug use, alcohol risk, and depression.

### Strengths and limitations

The study involved purposive sampling of practices, and recruitment of a sample that was a reasonable approximation of patients encountered regularly in primary care. However, the number of practices was small and participants were not randomly sampled, while data on response rates were not recorded. Findings may be affected by refusals to participate and missing data, which were high for the gambling problem measure. This comprised the PGSI,^22^ which does not assess the full breadth of gambling-related harms.^26^ To reduce burden, the study used a single-item measure of gambling problems among family members, while clinical characteristics were measured using brief screens that possess moderate specificity,^20^^,^^27^ and do not correspond to severe mental health concerns and addiction problems.

### Comparison with existing literature

Estimates of the extent of gambling problems were lower than those in prior research from the US,^13^^,^^14^ and are similar to levels in population-based studies in the UK.^1^ Notwithstanding, the present findings highlight that gambling problems are important clinical issues for primary care attenders, that are strongly linked with poor mental health^9^ and have impacts that extend beyond the individual.^28^ There is evidence that people with gambling problems can benefit from therapeutic interventions, including intensive and brief interventions,^29^^,^^30^ and alongside minimal interventions for ‘concerned significant others’.^31^ These provide the basic components of an intervention framework that aligns with models of care for alcohol misuse, and comprises multiple tiers of intervention.^32^ These address a spectrum of severity (for example, simple advice or brief interventions for hazardous or harmful drinking, intensive therapies for dependence), as well as support needs of families, and have bases in identification strategies that are situated within primary care.^11^

### Implications for research and practice

The study indicates that around one in 20 patients report some degree of gambling problem in routine primary care, and highlights the need for improved acknowledgement and capacities to respond to these issues. It supports the recommendation that GPs and clinical staff should be vigilant for gambling problems,^12^ and particularly among young males and patients who are depressed or using alcohol and drugs. At a minimum, there should be training and support for clinical staff in identification and pathways to care. In the absence of visible signs of gambling problems that are low to moderate in severity, however, it seems unlikely that such strategies (which exclude questioning in the absence of visible risk factors) will identify many individuals who would benefit from early intervention. As such, it may also be that selective screening^33^ of high-risk groups (for example, young males and/or patients with depression), or within particular contexts (for example, university clinics), is potentially appropriate.

There is need for further evidence that indicates the burden of gambling problems in primary care at a national level, particularly illustrating co-occurrence and impact on other presenting problems. The development of strategies to identify gambling problems is associated with particular research needs, including studies which demonstrate that initiatives can yield improved access to interventions, and also that patients in primary care, who are not seeking help for gambling, will benefit from interventions.

Finally, these identification strategies can be justified only if adequate services are available to deliver interventions. It appears that such requirements are lacking in the UK, where intervention research for gambling is virtually non-existent, while treatment services are grossly inadequate.^34^ Such inadequacies are notwithstanding the best efforts of service providers (which mainly comprise voluntary sector organisations), and can be attributed to an unusual situation in the UK whereby research and treatment are commissioned almost exclusively by gambling industry-affiliated bodies. Given that between 15% and 40% of most gambling revenues (depending on type of activity),^35^ is derived from people reporting problems with gambling in the UK, there are conflicts of interest between public health and economic policy goals (whereby even small reductions in numbers of people gambling heavily implies far larger reductions in economic yield).^36^ Because of the vested interests of addiction industries,^36^^,^^37^ evidence and interventions that are supported through independent funding are needed.

Gambling should be formally recognised as a health-related issue in the UK, and included within the remits of mainstream commissioning bodies that are responsible for public health and service provision.

## References

[1] Wardle H, Griffiths MD, Orford J (2012). Gambling in Britain: A time of change? Health implications from the British Gambling Prevalence Survey 2010. Int J Ment Health Addict.

[2] Ofcom (2013). Trends in advertising activity — Gambling.

[3] Delfabbro P (2013). Problem and pathological gambling: a conceptual review. J Gambl Bus Econ.

[4] World Health Organization (1992). The ICD-10 classification of mental and behavioural disorders Clinical descriptions and diagnostic guidelines.

[5] American Psychiatric Association (2013). Diagnostic and statistical manual of mental disorders. 5th edition (DSM–5).

[6] Wardle H, Seabury C, Craig R, Mindell J (2013). Section 7 Gambling behaviour. Health Survey for England 2012 Volume 1 Health, social care and lifestyles.

[7] Orford J, Wardle H, Griffiths M (2010). The role of social factors in gambling: Evidence from the 2007 British Gambling Prevalence Survey. Community Work Fam.

[8] Goodyear-Smith F, Arroll B, Kerse N (2006). Primary care patients reporting concerns about their gambling frequently have other co-occurring lifestyle and mental health issues. BMC Fam Pract.

[9] Cowlishaw S, Kessler D (2016). Problem gambling in the UK: implications for health, psychosocial adjustment and health care utilization. Eur Addict Res.

[10] Evans L, Delfabbro PH (2005). Motivators for change and barriers to help-seeking in Australian problem gamblers. J Gambl Stud.

[11] O’Flynn N (2011). Harmful drinking and alcohol dependence: advice from recent NICE guidelines. Br J Gen Pract.

[12] George S, Gerada C (2011). Problem gamblers in primary care: can GPs do more?. Br J Gen Pract.

[13] Pasternak AV, Fleming MF (1999). Prevalence of gambling disorders in a primary care setting. Arch Fam Med.

[14] Morasco BJ, Vom Eigen KA, Petry NM (2006). Severity of gambling is associated with physical and emotional health in urban primary care patients. Gen Hosp Psychiatry.

[15] Petry NM, Blanco C (2013). National gambling experiences in the United States: will history repeat itself?. Addiction.

[16] Whooley MA, Avins AL, Miranda J, Browner WS (1997). Case-finding instruments for depression. Two questions are as good as many. J Gen Intern Med.

[17] Kroenke K, Spitzer RL, Williams JB, Monahan PO, Löwe B (2007). Anxiety disorders in primary care: prevalence, impairment, comorbidity, and detection. Ann Intern Med.

[18] Kendrick T, Pilling S (2012). Common mental health disorders — identification and pathways to care: NICE clinical guideline. Br J Gen Pract.

[19] Bradley KA, DeBenedetti AF, Volk RJ (2007). AUDIT-C as a brief screen for alcohol misuse in primary care. Alcohol Clin Exp Res.

[20] Rumpf HJ, Hapke U, Meyer C, John U (2002). Screening for alcohol use disorders and at-risk drinking in the general population: psychometric performance of three questionnaires. Alcohol Alcohol.

[21] McNeely J, Cleland CM, Strauss SM (2015). Validation of Self-Administered Single-Item Screening Questions (SISQs) for unhealthy alcohol and drug use in primary care patients. J Gen Intern Med.

[22] Ferris J, Wynne H (2001). The Canadian problem gambling index.

[23] Williams RJ, Volberg RA (2014). The classification accuracy of four problem gambling assessment instruments in population research. Int Gambl Stud.

[24] Salonen AH, Castrén S, Alho H, Lahti T (2014). Concerned significant others of people with gambling problems in Finland: a cross-sectional population study. BMC Public Health.

[25] Li E, Browne M, Rawat V (2017). Breaking bad: comparing gambling harms among gamblers and affected others. J Gambl Stud.

[26] Langham E, Thorne H, Browne M (2016). Understanding gambling related harm: a proposed definition, conceptual framework, and taxonomy of harms. BMC Public Health.

[27] Bosanquet K, Bailey D, Gilbody S (2015). Diagnostic accuracy of the Whooley questions for the identification of depression: a diagnostic meta-analysis. BMJ Open.

[28] Cowlishaw S, Suomi A, Rodgers B (2016). Implications of gambling problems for family and interpersonal adjustment: results from the Quinte Longitudinal Study. Addiction.

[29] Cowlishaw S, Merkouris S, Dowling N (2012). Psychological therapies for pathological and problem gambling. Cochrane Database Syst Rev.

[30] Yakovenko I, Hodgins DC (2016). Latest developments in treatment for disordered gambling: review and critical evaluation of outcome studies. Curr Addiction Rep.

[31] Hodgins DC, Toneatto T, Makarchuk K (2007). Minimal treatment approaches for concerned significant others of problem gamblers: a randomized controlled trial. J Gambl Stud.

[32] Department of Health (2006). Models of care for alcohol misusers (MoCAM).

[33] O’Doherty LJ, Taft A, Hegarty K (2014). Screening women for intimate partner violence in healthcare settings: abridged Cochrane systematic review and meta-analysis. BMJ.

[34] George S, Copello A (2011). Treatment provision for Britain’s problem gamblers: Present gaps and future opportunities. Adv Psychiatr Treat.

[35] Orford J, Wardle H, Griffiths M (2013). What proportion of gambling is problem gambling? Estimates from the 2010 British Gambling Prevalence Survey. Int Gambl Stud.

[36] Adams PJ (2016). Moral jeopardy Risks of accepting money from tobacco, alcohol and gambling industries.

[37] Babor T, Hall W, Humphreys K (2013). Who is responsible for the public’s health? The role of the alcohol industry in the WHO global strategy to reduce the harmful use of alcohol. Addiction.

